# The Passion Flower

**Published:** 1885-07

**Authors:** 


THE PASSION FLOWER.—According to Dr. George W. Winterburn, the therapeutic uses of the white passion flower resemble the bromides on one hand and gelsimium on the other. It is one of our best hypnotics, producing a quiet, pleasant sleep, altogether different from the comatose stupor of morphia, and from which the patient may be aroused at any moment. It may be given in doses of two or three drops of the tincture or low dilution. Even in the worst form of sleeplessness, that associated with suicidal mania, this drug will produce quiet slumber, from which the patient awakes with clear mind and rational thoughts. In its control of convulsion, passiflora closely resembles gelsemium. It will be found of service in opisthotonos, trismus and tetanus.—*Amer. Homœopath.*

